# Late gadolinium enhancement by cardiovascular magnetic resonance provides prognostic information in symptomatic alcoholic cardiomyopathy

**DOI:** 10.1186/1532-429X-17-S1-P316

**Published:** 2015-02-03

**Authors:** Wei Xiangli

**Affiliations:** 1 Department of Radiology, Fuwai Hospital, National Center for Cardiovascular Diseases, Chinese Academy of Medical Sciences and Peking Union Medical College, Beijing, China

## Background

Previous studies have demonstrated that late gadolinium enhancement (LGE) on cardiovascular magnetic resonance (CMR)predicted independently cardiac adverse outcomes in dilated cardiomyopathy (DCM). ACM patients did not have a better outcome than DCM and prognosis was poorer after development of heart failure. There is no report about the association between alcoholic cardiomyopathy (ACM) outcomes and LGE.

## Methods

70 consecutive ACM patients from July 2008 to December 2010 were examined with LGE during CMR. The mean follow-up time was 42.3±17.5 months after CMR. Cardiac events include cardiac death, implantable cardioverter-defibrillator (ICD) discharge, hospitalisation for decompensated congestive heart failure (CHF) and heart transplantation.

## Results

Of the 70 patients, myocardial fibrosis visualized by LGE was detected in 24(34.3%) patients. During the follow-up period, most events (n=9) were related to hospitalisation for decompensated CHF in the total 13 cardiac events. The incidence of cardiac events was significantly higher in patients with LGE than that without LGE (37.5% vs. 8.7%, P=0.003). When entered into multivariate Cox regression analysis, the presence of LGE yields hazard ratio (HR) of 4.62 (95% CI, 1.4 to 15.4) for cardiac events (P=0.007), the extent of LGE also retains its independent predictive value in LGE (+) patients with HR of 1.13 (95% CI, 1.04 to 1.22, P =0.003).

## Conclusions

In patients with symptomatic ACM, the presence of LGE determined by CMR is a strong independent predictor of adverse cardiac events.

## Funding

This study was supported by grant No. 81130029 from the key projects of National Natural Science Foundation of China and by grant (Z121107001012114) from Peking Science and Technology Commission.

